# Bone marrow derived stem cells (BMSC) may improve memory in Alzheimer’s Disease

**DOI:** 10.1002/alz.083902

**Published:** 2025-01-09

**Authors:** Jeffrey Neill Weiss, Steven Levy

**Affiliations:** ^1^ Parkland, FL USA; ^2^ Westport, CT USA

## Abstract

**Background:**

A 73‐year‐old female with a 3 year history of Alzheimer’s disease was treated within the protocol of The Alzheimer’s Autism and Cognitive Impairment Stem Cell Treatment Study (ACIST), an IRB approved clinical study registered with clinicaltrials.gov NCT 03724136.

**Method:**

The procedure consists of bone marrow aspiration, cell separation using an FDA cleared class 2 device, and intravenous and intranasal administration of the stem cell fraction. The patient’s son, a physician, recorded a video on the day prior to surgery, and on the first postoperative day.

**Result:**

1 Day Pre‐Surgery https://drive.google.com/file/d/1fTfceevWLpPSh4J4V6XGenx3bMwNzEBp/view?usp=drive_web 1 Day Post‐Surgery https://drive.google.com/file/d/14AhVTes2kQ50tWzj6eWE5fEamgEJWa_D/view?usp=drive_web

The improvements lasted several weeks before gradually regressing.

**Conclusion:**

Potential mechanisms of action of bone marrow derived stem cells (BMSC) are varied. These may include: the release of neurotrophic factors (NTF) including ciliary neurotrophic factor which has been shown to alleviate excitotoxicity in neurons; the release NGF (nerve growth factor) which enhances neuronal survival and function; and, immune modulation in a setting where neuroinflammation may exacerbate damage. BMSC can transfer their own organelles, including mitochondria and lysosomes, to injured or stressed cells, as well as release exosomes (secretome) containing proteins, mRNA and miRNA, which have the ability to regenerate axons and address neuronal dysfunction.

Human mesenchymal cells (hMSC), a subpopulation of BMSC, have been reported to provide de novo neuron regeneration. BMSC, through fusion with Muller cells (a retinal specific glial cell) can lead to new ganglion cells. Potentially, there may be local activity of BMSC directly in the hippocampus to help clear exogenous amyloid deposits, reduce microglial immunologic activity and enhance axonal recovery, including dendritic recovery and activity.

The improvement of long‐term memory in this patient suggests that neuron connectivity remains in place, for long periods after diagnosis, and can potentially be recovered.

Future research should include isolating components responsible for the improvement with the hope of extending the duration of response.

